# Insights into the Phytochemical Composition and Bioactivities of Seeds from Wild Peony Species

**DOI:** 10.3390/plants9060729

**Published:** 2020-06-09

**Authors:** Zhenguo Yan, Lihang Xie, Yao Tian, Mengchen Li, Jing Ni, Yanlong Zhang, Lixin Niu

**Affiliations:** 1 College of Landscape Architecture and Arts, Northwest A&F University, Yangling 712100, Shaanxi, China; yanzhenguo@nwafu.edu.cn (Z.Y.); xielihang@nwafu.edu.cn (L.X.); tianyao@nwafu.edu.cn (Y.T.); limengchen@nwafu.edu.cn (M.L.); nijing@nwafu.edu.cn (J.N.); 2 Oil Peony Engineering Technology Research Center of National Forestry Administration, Yangling 712100, Shaanxi, China

**Keywords:** peony species, seed, phytochemical characterization, bioactivity evaluation, paeoniflorin

## Abstract

Peony is an important medicinal and ornamental plant widely cultivated in the world. Its seeds as a functional food source have attracted much more attention in recent years, and they are rich in monoterpene glycosides and phenolic compounds. To assess the application value of wild peony seeds, the main chemical composition and content, such as total phenolic content (TPC), total flavonoid content (TFC), total flavanol content (TAC), and α and γ tocopherol content, of the seeds from 12 species and 2 subspecies were systematically explored in the present study. Four different antioxidant assays (DPPH, ABTS, FRAP, and HRSA), antibacterial, and antifungal assays were also performed using various in vitro biochemical methods. The results showed that the seeds of *P. delavayi*, *P. obovata.* subsp*. obovata,* and *P. rockii.* subsp*. rockii* had a high content of TPC, TFC, and TAC, respectively. Twenty-five individual chemical compounds were qualitatively and quantitatively measured by HPLC-MS, with paeoniflorin being the most abundant compound in all samples. *P. mairei* was grouped individually into a group via hierarchical cluster analysis according to its relatively highest monoterpene glycosides and TPC content. This work has provided a basis for the development and utilization of seeds for the selection of wild peony species of dietary interest.

## 1. Introduction

Peony is an important medicinal plant in China and is recorded in the Pharmacopoeia of the People’s Republic of China. The root of the peony is well known as “Baishao”, “Chishao”, and “Danpi”, and has been used for the treatment of dementia and blood stagnation syndrome and as an antihyperglycemic, analgesic, anti-inflammatory, and antispasmodic agent [1,2]. Because of its high ornamental value, the peony is also an important ornamental flower and is widely planted in China, Japan, Europe, and the United States [3]. In addition, there are 33 species and 25 subspecies belonging to the genus *Paeonia L.* (Paeoniaceae), including 3 sections, namely, sect *Moutan* DC., sect. *Onaepia* Lindl., and sect. *Paeonia*. Moreover, a total of 15 species and 5 subspecies are widely distributed in China, where is the natural distribution center of the genus *Paeonia* [4].

Peony seeds have attracted much more attention in recent years because they are rich in unsaturated fatty acids (linolenic acid, linoleic acid, oleic acid, and palmitic acid) [5,6]. The α-linolenic acid was the most abundant fatty acid in the seed oil of *P. veitchii,* comprising 36.14% of total fatty acids by weight [7]. It is higher than that in camellia (10%), olive (8%), and avocado (10%) oils [8]. Yang et al. reported that unsaturated fatty acids *P. decomposita* accounted for 91.94–93.70% by weight, while the α-linolenic acid content was 40.45~47.68% of total fatty acids by weight [9]. Their results showed that herbaceous peony seed oil was rich in unsaturated fatty acids and γ-tocopherols [10]. Additionally, bioactive phytochemicals, such as phenolic compounds, flavonoids, flavanols, monoterpene glycosides, and oligostilbenes, were also found in peony seeds [11]. The seeds of *P. rockii* and *P. decomposita* subsp*. rotundiloba* are also regarded as promising sources of natural antioxidants [12]. Oidovsambuu et al. analyzed the fruit of *P. anomala*, which is rich in methyl gallate, albiflorin, and paeoniflorin [13]. The roots of peony cultivated production cycles about 4–5 years, a large number of seeds are produced after the third year and those become important agricultural by-products. The use of these large amounts of seeds not only increases the economic value of peonies but also helps to deal with agricultural wastes for sustainable development. Therefore, it is necessary to develop the utilization of peony seeds as high value-added products.

However, previous studies mostly focused on limited peony species. As important development materials for germplasm resources, wild peony species native to China have not been well studied. Moreover, the reports on the antioxidant and antibacterial properties of peony seeds are scarce.

This research aims to exploit the developing potential of peony seeds for applications in the food and pharmaceutical industries. To guide a comprehensive evaluation, the seeds from 12 species and 2 subspecies of wild peonies native to China were systematically explored regarding their phytochemical components in this study. Four different antioxidant assays were employed for antioxidant activity evaluation. In addition, the total phenolic content (TPC), total flavonoid content (TFC), total flavanol content (TAC), and the α- and γ-tocopherol content were measured, and the antibacterial and antifungal activities of the extracts were also determined.

## 2. Results and Discussion

### 2.1. TPC, TFC, and TAC of Peony Seeds

**Figure 1:**
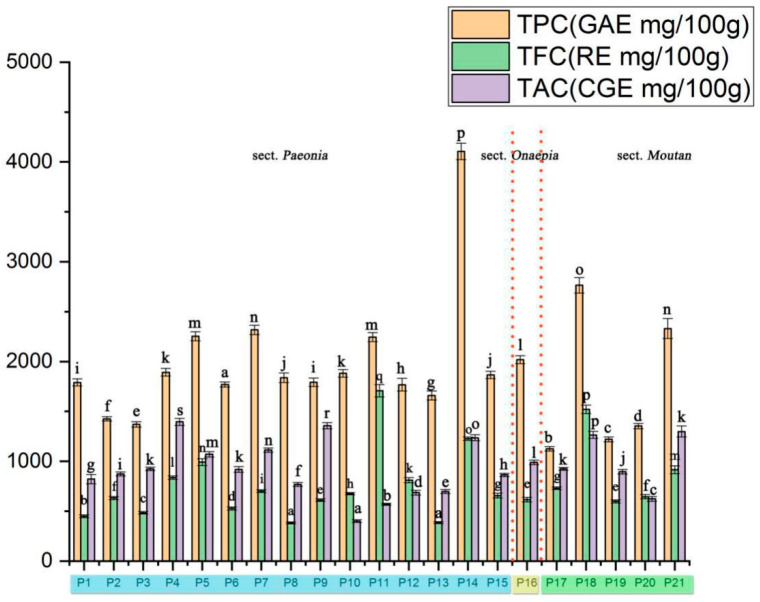
TPC, TFC, and TAC contents of seed extracts from different peony species. Different letters on bars indicate significant difference at *p* ≤ 0.05 by using Duncan’s test.

The TPC, TFC, and TAC in the seeds from 21 populations were presented in Figure 1.

The range of the TPC was from 1126.04 (P17) to 4105.5 mg GAE/100 g (P14), which was higher than that in pomegranate seeds (165 mg GAE/L), sesame seed oil (26.00 ± 0.14 mg GAE/g), and orange seeds (230.4 mg GAE/kg) [14,15]. These values were close in range to those obtained for flowers from different Zhongyuan tree peony cultivars (3.97 to 21.73 GAE/g) [16]. In contrast, the TPC of leaves from *P. rockii* was considerably lower than that observed in our research. Based on the data, the TPC in sect. *Paeonia* was higher than that in the sect. *Moutan*. This may be directly related to the genetic characteristics and material distribution characteristics of peony seeds. The accumulation of phenolic substances may be affected by the photosynthesis intensity of the plant leaves, which is different due to genetic differences. Different extraction methods also have certain effects on TPC; the experiment revealed ethanol showed the high TPC content of apple [17].

The TFC of the peony seed varied from 382.52 (P8) to 1707.37 (P11) mg CGE/100 g DW. This result is similar to that of tree peonies reported by Zhang et al. [5]. Some other studies also revealed that the TFC of flowers from tree peony species was higher than that in our research [18]. The distribution of flavonoids in plants is unbalanced; simple polyphenols, such as ketones and ketols, are mainly found in the stem and leaf organs of plants, while anthocyanins and tannins are mainly found in the flower and fruit organs [19].

Flavanols, a subgroup of the flavonoid family, represent a major group of flavonoids in the Western diet [20]. The TAC was significantly different in different samples, ranging from 401.67 (P10) to 1395 mg RE/100 g DW (P4). The TAC ranges of cocoas (up to 920–1220 mg/100 g), apples (up to 120 mg/200 g), and tea (up to 300 mg/infusion) were reported by Mafuvadze et al. [21].

Overall, these findings suggest that seeds from sect. *Paeonia* may be the better source to obtain extracts with TPC, and several species (P18 and P21) could be developed as natural sources for both flavonoid and flavanol production.

### 2.2. The Main Phytochemical Components of the Peony Seeds

**Figure 2:**
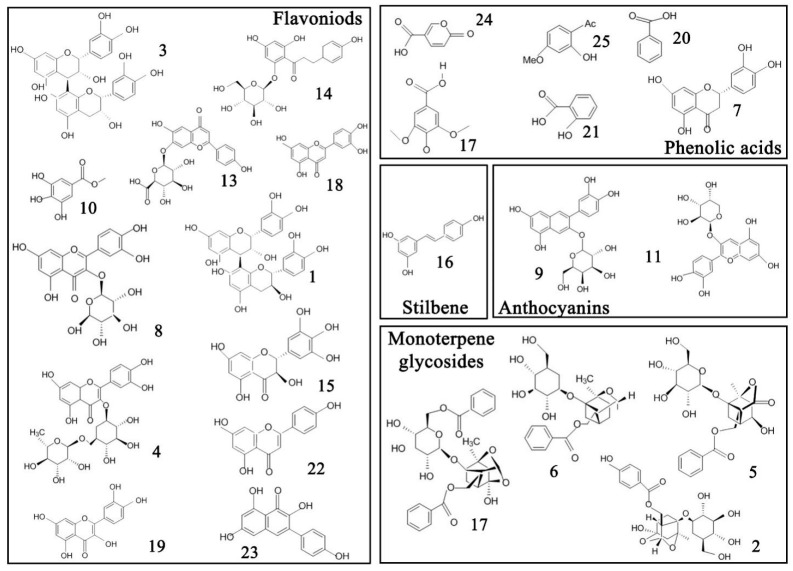
Chemical structures of the 25 common metabolites identified in the seeds of peony species.

**Figure 3:**
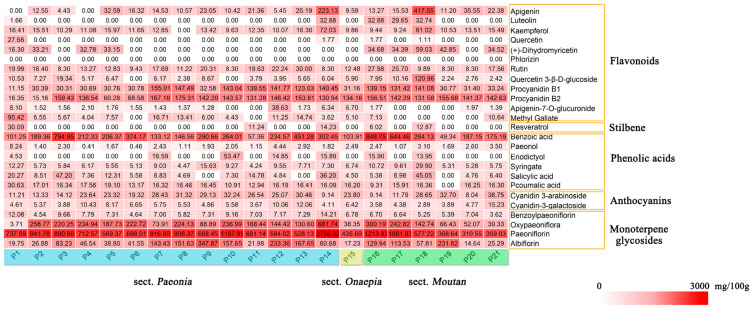
Concentrations (mg/100 g DW) of metabolites and compounds identified from seed kernel and seed shell extracts of 21 populations of peony species (*n* = 3).

HPLC-MS was used for the simultaneous determination of the phytochemical component content of the seeds of various peony species. The main components of the seed extracts from different peony species are shown in Table 1 and Figure 2 and Figure 3. The identified components include two flavanones, twelve flavonoids, one stilbene, six phenolic acids, two anthocyanins, and four monoterpene glycosides. There were highly significant differences between the relative content of the proximate composition and phytochemical components among the peony seed extracts.

Procyanidin B2 was detected in the seed extracts of all peony species, with content ranging from 15.16 to 175.31 mg/100 g DW. Procyanidins belong to flavonoids, which are widely present in fruits (especially in cocoa, grape, and berries), and the procyanidin B2 is the most widely distributed type. Procyanidin oligomers have been shown to have potent antioxidant activity [22,23]. This is the first time that procyanidin has been found in the seeds of peony.

The most abundant identified phenolic acid in the seeds was benzoic acid, with its concentration ranging from 49.34 (P19, sect. *Moutan*) to 848.75 (P16, sect. *Moutan*) mg/100 g DW. Benzoic acid has shown high potential as an antifungal compound and is also used as a preservative [24]. The content of syringate, salicylic acid, and p-coumalic acid in the seeds was similar, ranging from 4.24 to 29.9, 4.69 to 47.2, and 9.31 to 30.63 mg/100 g DW, respectively. Anthocyanins are natural water-soluble pigments widely found in plants [25]. Two anthocyanins (cyanidin 3-arabinoside and cyanidin-3-galactoside) were detected in most of the investigated samples, and their concentrations were relatively low. Cyanidin 3-arabinoside is a strong antioxidant in emulsions and in low-density lipoproteins in humans [26].

Monoterpene glycoside is the most important bioactive compounds in the genus *Paeonia L* and is characteristic of the genus. Paeoniflorin, a major monoterpene glycoside, is the main active ingredient in medicinal materials of the genus *Paeonia* [27]. Since Shibata first isolated paeoniflorin from the peony roots in 1963, it has been widely studied as an antioxidant and anticonvulsant [28]. Simultaneously, the discovery of paeoniflorin has also become an important basis for the botanical classification of the genus *Paeoniae* [29]. Accordingly, paeoniflorin is an important indicator for evaluating the phytochemical properties of peony seeds. Our results showed that paeoniflorin was the most abundant monoterpene glycoside in all extracts and its content ranged from 310.55 to 2756.92 mg/100 g DW. The paeoniflorin content in peony seeds was lower than that in the wild radix Paeoniae Rubra (34.50–85.00 mg/g) [30]. Paeoniflorin content in seeds of most sect. *Paeonia* species is higher than that of sect. *Moutan*, while content in sect. *Onaepia* is between the two species. The synthesis of terpenoids in plants is greatly affected by environmental temperature with high sensitivity. Different types of terpenoids are affected differently by the environment and the low-temperature environment is beneficial to the synthesis of nonvolatile terpenoids [31]. By comparison, it was found that the distribution range of the sect. *Paeonia* was wider, the latitude was lower in the three sections, and it faced more extreme low temperature. Therefore, the rich paeoniflorin content may also be a response mechanism to the environment. At the same time, the paeoniflorin content of the *P. delavayi* (P16 and P17) belonging to sect. *Moutan* is also higher, which faces extreme minimum temperature of −16 °C. Therefore, the role of paeoniflorin in resistance to low temperature stress in Paeoniaceae deserves attention. The oxypaeoniflora content ranged from 38.35 (P15) to 681.74 mg/100 g DW (P14), making it the second most abundant monoterpene glycoside in the extracts. The biosynthesis pathway of monoterpenoids is mainly the isoprene pathway, so the synthetic pathways of paeoniflorin and oxypaeoniflora are similar. It is speculated that the difference in the content of the two is related to their stability or transport mode [32]. The results showed that the seed was an important natural resource of a relatively large amount of monoterpene glycosides, which has great development potential for food and medicinal purposes, especially those of *P. mairei* and *P. stemiana*.

In general, the relative content of procyanidin B2, benzoic acid, paeoniflorin, and oxypaeoniflora was higher than those of other components in the seed.

### 2.3. Tocopherols (α and γ-Tocopherol) of Peony Seeds

**Figure 4:**
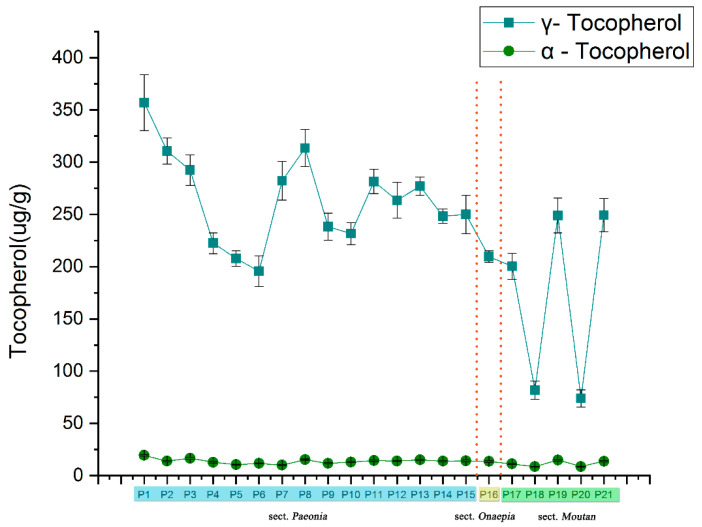
Determination of tocopherols (α and γ tocopherol (μg/g)) of seed extracts from different peony species.

The α- and γ-tocopherol content were determined in all samples by HPLC, as shown in Figure 4. γ-tocopherol was the dominant component in the kernel extracts, with its concentration ranged from 73.91 (P20) to 357.01 (P1) µg/g DW. Previous studies suggest that γ-tocopherol is important to human health [33]. However, humans and animals do not synthesize γ-tocopherol; moreover, γ-tocopherol is highly abundant in plant seeds. However, the α-tocopherol content was low in the extracts, and the highest content was found in P1 at only 19.32 µg/g. The results agreed with previous reports that the average γ-tocopherol content in herbaceous peony seed oil was 337.47 ± 57.42 µg/g, accounting for approximately 92.97% of the total tocopherol content [10]. The results showed that the seeds of *P. lactiflora* and *P. anomala* subsp. *anomala* might be further developed as a dietary source of vitamin E.

### 2.4. Assessment of Antioxidant Activities

**Figure 5:**
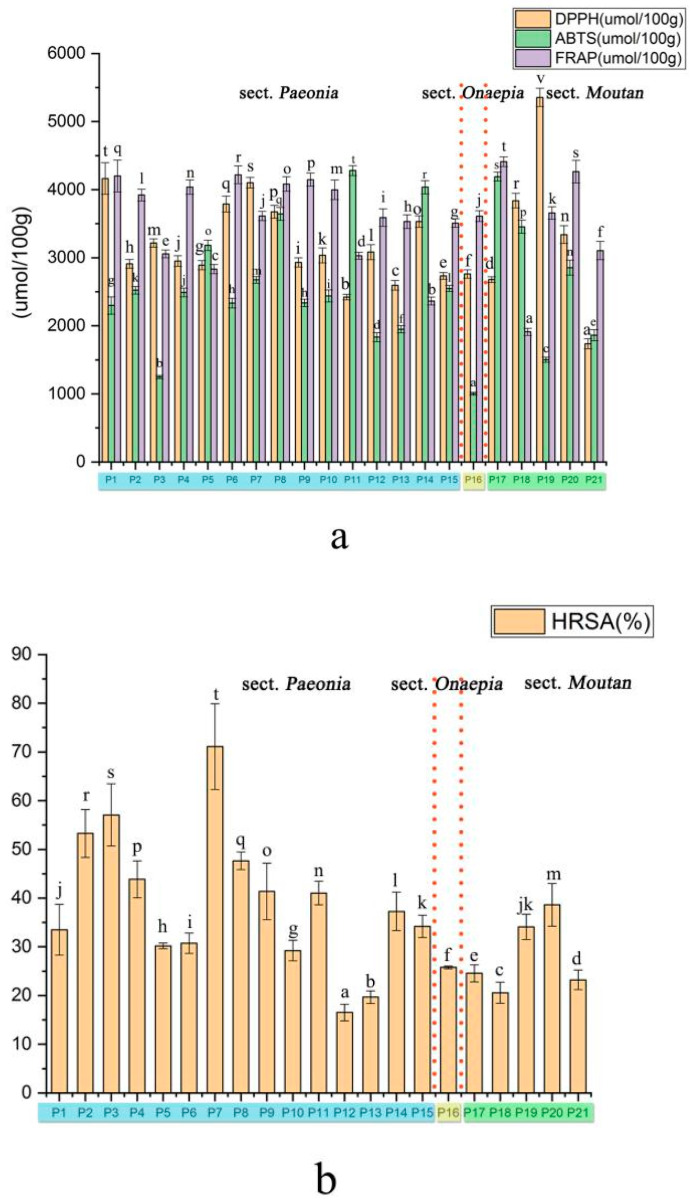
Antioxidant activities of seed extracts from different peony species. (**a**)DPPH, ABTS, FRAP (**b**) HRSA. Different letters on bars indicate significant difference at *p* ≤ 0.05 by using Duncan’s test.

Oxidative stress induces changes and lesions in DNA, causing aging and diseases [34]. Foods can provide antioxidants to directly prevent oxidation in humans [35]. Therefore, this study aimed to evaluate the antioxidant activity of peony seeds. Some substances in vitro antioxidant activity have the same trend as in vivo, and therefore, some techniques in vitro have been used to allow rapid screening of substances about the antioxidant activity. In our research, all samples showed various antioxidant properties (DPPH, ABTS, FRAP, and HRSA). The results of the antioxidant activity assays are presented in Figure 5.

The DPPH method assesses the scavenging and reduction of the DPPH radical to determine the antioxidant capacity [36]. The antioxidant activity of all seeds ranged from 5354.67 (P19, sect. *Moutan*) to 1737.33 (P21, sect. *Moutan*) mmol TE/100 g DW. Similar results were found by HE et al. [37]. The ABTS radical scavenging capacity of the extracts varied from 4281.18 to 173.22 μmol TE/100 g DW, and the average level of ABTS radical scavenging was 3225.65 μmol TE/100 g DW. In addition, the leaf of *P. ostii* had significant effects on ABTS radical scavenging ability [38]. Currently, FRAP methods are widely used to determine the reducing power of antioxidants by iron reduction. The FRAP values of the extracts ranged from 4411.63 to 1911.51 mmol/100 g DW. Among the peony species, the seeds from *P. delavayi* (P17) showed the strongest FRAP radical scavenging capacity; the sample also had a high content of benzoic acid and procyanidin B2, both of which have shown antioxidant properties. There were significant differences among the HRSA results for the samples. Among the evaluated species, P7 showed the greatest radical scavenging effect, and the lowest effect was observed for P12. In some studies of wild tree peony seed kernels, the HRSA was determined to be 19.24% (*P. lutea*) to 42.47% (*P. decomposita*), which agreed with the results of our research [5]. Different populations of the same species also showed a difference in HRSA: P1 (55.07%), P2 (53.26%), P3 (33.5%), P7 (61.09%), and P8 (47.65%). Peony seeds are rich in polyphenols and stilbenoid compounds, which may be responsible for their antioxidant activity. Naturally, the content of stilbenoid compounds is low, and there is no effective synthesis method for them. Therefore, peony seeds could be developed as an important plant resource for the isolation of stilbenoid compounds. The correlation between the synergistic, antagonistic, and antioxidant activities of phenolic substances in peonies should be determined in future studies.

### 2.5. Antibacterial and Antifungal Activities of Peony Seeds

**Figure 6:**
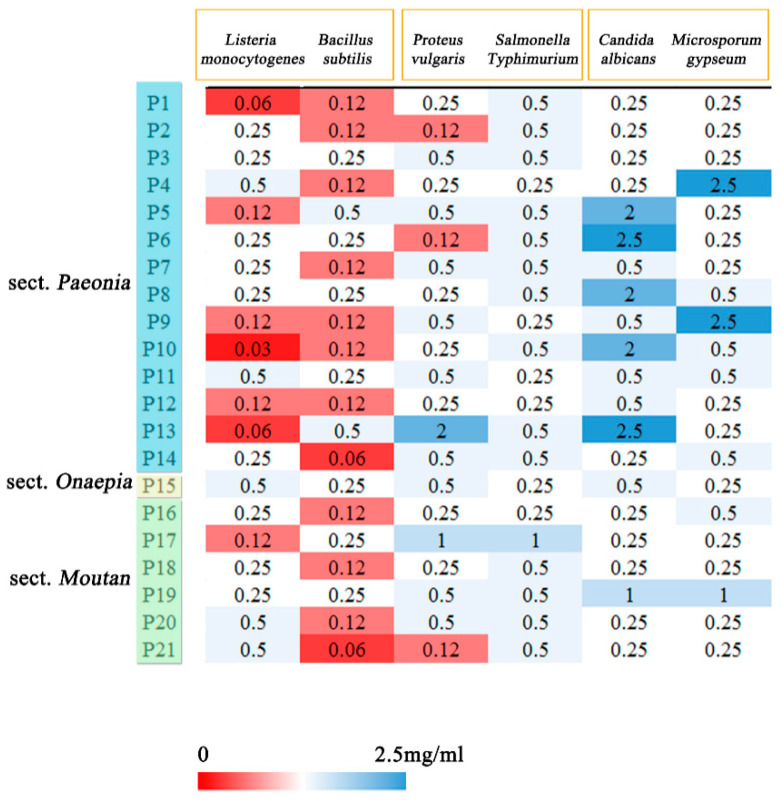
Antimicrobial activities (MIC mg/mL) of seeds extracts from different species.

**Figure 7:**
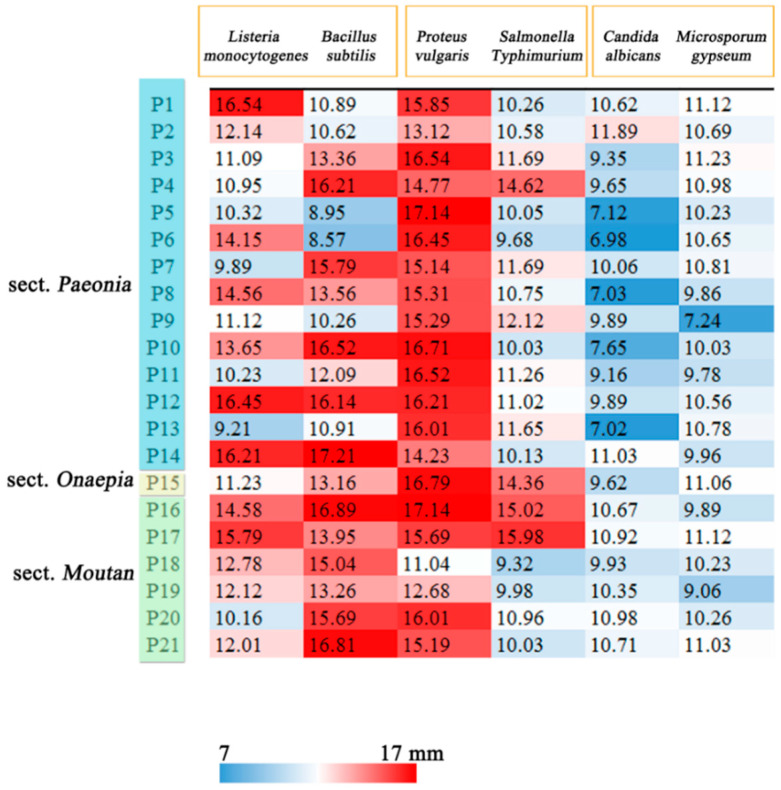
Antimicrobial activities (IZR mm) of seeds extracts from different species.

Bacterial resistance is considered as a global public health problem and is necessary for bacteriostasis, therefore, the antibacterial and antifungal activities of extracts were determined. All samples exhibited antimicrobial activities against Gram-positive and Gram-negative bacteria and fungi at different levels. The MIC and IZR values for antimicrobial activity of the extract are listed in Figure 6 and Figure 7 (Details in supplementary Tables S1 and S2). The seed extracts showed strong activity against Gram-positive bacteria (*Listeria monocytogenes* and *Bacillus subtilis*); the MIC ranged from 0.03 to 0.5 mg/mL, and the IZR ranged from 8.57 to 17.14 mm. The *Listeria monocytogenes* is the major foodborne pathogen [39]. Sun et al. found that the MIC value of blueberry extract was 0.53 mg/mL; it is obvious that peony seeds have a better antibacterial effect than blueberries [40]. All samples showed relatively weak growth inhibition of fungi (*Candida albicans* and *Microsporum gypseum*). For all extracts, the IZR had diameters less than 12 mm. In our research, the samples P12 and P14 showed stronger antibacterial activities against *Listeria monocytogenes* and *Bacillus subtilis* than those of the other samples.

Paeoniflorin, as a unique chemical substance in peony plants, showed strong antibacterial effects [41]. Additionally, the polysaccharide obtained from peony seed dregs showed antibacterial activity against both Gram-positive (*B. subtilis* and *S. aureus*) and Gram-negative (*E. coli* and *S. typhimurium*) bacteria [42]. There are abundant polyphenols in the seeds of wild peony plants [21]. These findings suggest that phenolic compounds have antimicrobial properties and prevent rancidity [43]. The concentration of benzoic acids in seed extract was also high and that displayed potent antibacterial activity [24].

Previously, the antibacterial and antifungal activities of peony seeds have not been intensively investigated and our research verified the effectiveness of seeds from wild peonies on controlling four pathogenic bacterial strains and two pathogenic fungi. Therefore, the seeds of peonies might be good potential prospects for the research and development of medicinal and food applications.

### 2.6. Relationships between Phytochemical Components and Bioactivities of Peony Seeds

**Figure 8:**
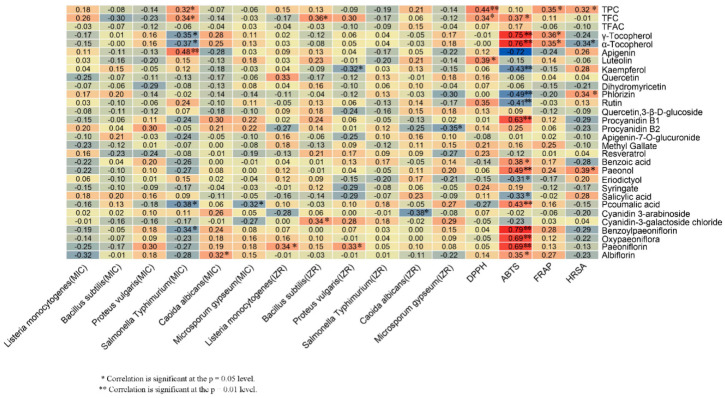
Relationships between phytochemical components and bioactivity activities.

Correlation analysis (CA) was conducted to address the relationships among the bioactivities and the phytochemical components of the samples (Figure 8). The TPC showed the highest correlation with DPPH at the *p* = 0.05 level and with both FRAP and HRSA at the *p =* 0.01 level. Abundant phenolic compounds in peony seeds enhanced the reducing power and significantly contributed to the antioxidant activity. The TFC also showed a significant correlation with the DPPH and ABTS radical scavenging abilities (*p* = 0.01 and *p* = 0.05, respectively), which was in accordance with the findings of Zhang et al. and Meng et al. [5,40]. Regarding antimicrobial activity, a significant correlation was observed for the TPC and TFC and the *Salmonella typhimurium* MIC and the *Bacillus subtilis* IZR at the *p* = 0.01 level.

Fifteen compounds were significantly correlated with the antioxidant capacity. There was a significant correlation between apigenin and the *Salmonella typhimurium* MIC (*p* = 0.05), between paeoniflorin and the *Proteus vulgaris* MIC, the *Listeria monocytogenes* IZR, and the *Proteus vulgaris* IZR at the *p* = 0.01 level, while procyanidin B1, paeonol, p-coumalic acid, oxypaeoniflora, and paeoniflorin showed correlations with the ABTS radical scavenging ability (*p* = 0.05). Previous phytochemical studies have revealed that the kaempfero isolated from *Bryophyllum pinnatum* showed antimicrobial and antioxidant properties [44]. Conversely, negative correlations were found between kaempferol and both the *Proteus vulgaris* IZR and the ABTS radical scavenging ability at *p =* 0.05 in this experiment. The p-coumalic acid was negatively correlated with the *Salmonella typhimurium* MIC and the *Microsporum gypseum* MIC at *p* = 0.01.

Synergistic activity between phytochemical components adjusts the interaction between bioactivity and complex mixtures present in different biological systems, and the activity can be significantly altered. The correlation between the phytochemical compositions and the bioactivities was effectively high in the mentioned cases. Additionally, it could be that other compounds also contribute to these activities. However, only 25 compounds were identified by us, this was a limitation in this experiment. Future research of peony seeds must carry out broad-targeted metabolomics and the correlations of bioactivity and monomeric substances.

### 2.7. Cluster and Principal Component Analysis

**Figure 9:**
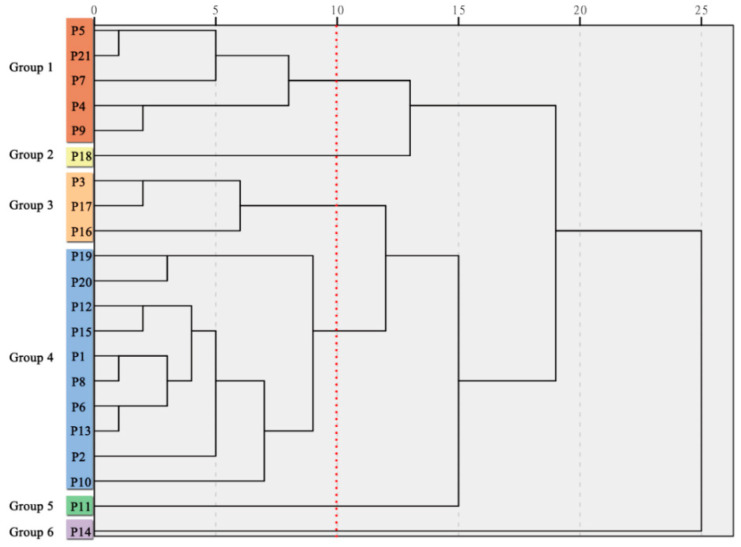
Dendrogram plot visualizing the clustering of 21 populations from peony based on the contents of the TPC, TFC, TAC, tocopherols, and 25 individual chemical compounds.

**Figure 10:**
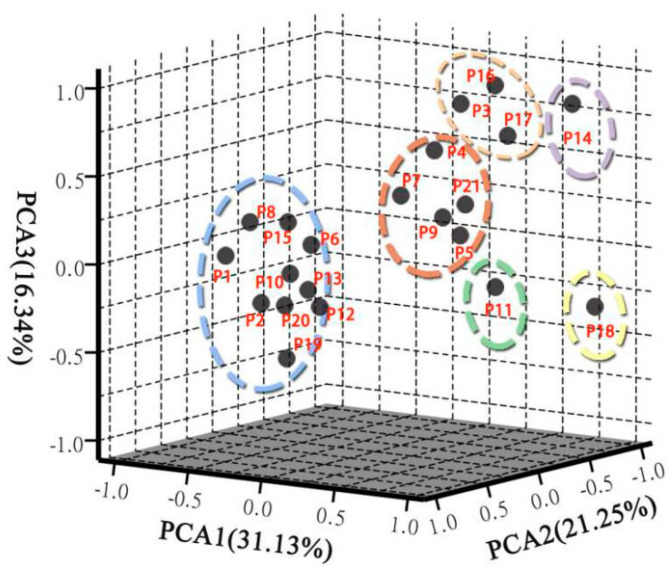
Principal component analysis for the 21 populations from peony based on the content of the TPC, TFC, TAC, tocopherols, and 25 individual chemical compounds.

**Figure 11:**
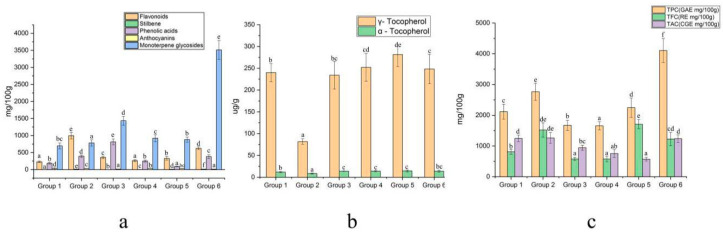
Variations in the content of total metabolite content TPC, TFC, TAC, five metabolite categories, and tocopherols between the six groups. (**a**) Flavonoids, Stilbene, Phenolic acids, Anthocyanins, Monoterpene glycosides. (**b**) γ-tocopherol, α-tocopherol. (**c**) TPC, TFC, TAC. Different letters on bars indicate significant difference at *p* ≤ 0.05 by using Duncan’s test.

The TPC, TFC, TAC, tocopherol content, and the content of 25 individual chemical compounds obtained from 21 populations were used to establish a hierarchical analysis by SPSS 21.0 software. In terms of the equipartition principle, the 21 populations were classified into 6 groups using Ward’s method (Figure 9). Principal component analysis (PCA) for the 21 peony populations based on the content of the TPC, TFC, TAC, tocopherols, and 25 individual chemical compounds were also established (Figure 10). The first three components had a great percentage (68.72%) of the total variance, and they were three-dimensionally plotted. PCA divided the 21 populations into six groups, including Group 1 (P4, P5, P7, P9, and P21), Group 2 (P18), Group 3 (P3, P16, and P17), Group 4 (P1, P2, P6, P8, P10, P12, P15, P19, and P20), Group 5 (P11), and Group 6 (P14). These results were similar to the sorting of HCA. Approximately 5 populations were grouped into Group 1, and 10 populations were grouped into Group 4; the two groups have no obvious advantage in phytochemical composition (Figure 11). P18 samples were grouped in Group 2, which contains the abundantly phenolic acids and TPC. P3, P17, and P16 were clustered into Group 3, which were characterized by high monoterpene glycosides content. P11 was divided into Group 5 with the highest γ-tocopherol. P14 samples were grouped into Group 6, which was characterized as having the highest monoterpene glycosides and TPC content. As important active compounds in peony plants, the oxypaeoniflora and paeoniflorin contents were highest in P14*,* with concentrations of 681.74 and 2756.92 mg/100 g DW, respectively. The species of *P. mairei* should deserve more attention in future research. However, there was no significant difference in the anthocyanin content between the groups.

Contrary to the fact that the fatty acids could be used as a chemotaxonomic tool for tree peony and herbaceous peony species, the results of clusters based on phytochemicals are not consistent with taxonomic results. [6,12]. Species in all three sections are not separated. The phytochemistry of peonies seeds is also affected by both genes and the environment.

The limitation is that only limited phytochemicals were analyzed in this article, which has a certain impact on the analysis results. To gain further insight into the evolution of peony, that broad-targeted metabolomics or genomics research could be applied, besides, the number of populations should be increased in sampling.

By comparing the geographical conditions of the six groups, we found that Group 4 was the most widely distributed one with the widest latitudinal and longitudinal distribution. Groups 5 and 6 have relatively similar geographic factors. In our previous research, we found that myristoleic acid in peony seed oil had a significant positive correlation with annual rainfall and latitude, and 1000-seed weight of peony seed was increased remarkably with the increasing latitude [6]. However, there is no obvious regularity between the three geographic factors and the phytochemicals. This may be limited to the fact that we only collected three environmental factors, and the population of each species is relatively small. In future research, we recommend collecting more detailed environmental factors and expanding the number of sampled populations.

## 3. Materials and Experimental Methods

### 3.1. Herbal Materials

**Figure 12:**
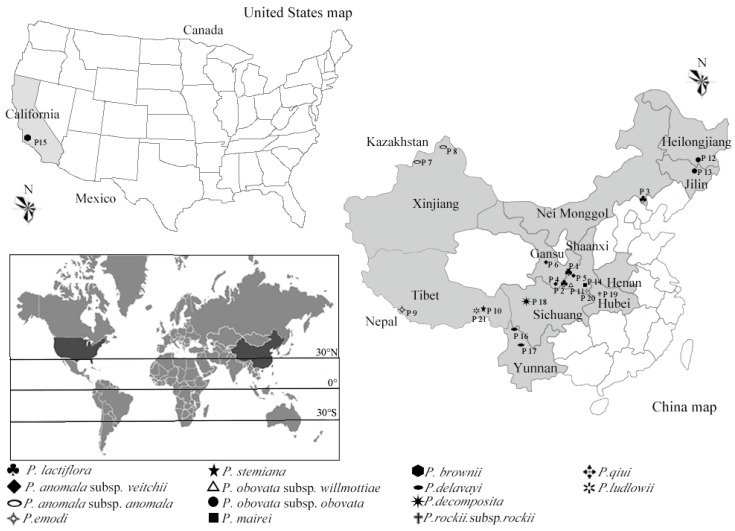
The natural habitat sites of 21 populations.

The peony seeds were collected from 21 populations, which are growing up in its natural habitats (China and America), in August and September of 2018 (Table 2 and Table 3 and Figure 12). Twenty plants were randomly selected from one population, and three fruits were picked from every plant. Every single sample was mixed by the fruits of each population. Seeds were hulled, and then the kernel was dried by a vacuum-freeze (BENCHTOP K, VIRTIS CO. Ltd., Warminster, PA, USA). The seeds were pulverized and stored at −20 °C without light for further studies.

### 3.2. Chemicals

Folin-Ciocalteu phenol reagent, gallic acid, Querc-3-O-rutinoside (rutin), and chemical standards (including α and γ-tocopherol) were purchased from Sigma-Aldrich. Trolox (97%), 2,2-diphenyl-1-picrylhydrazyl (DPPH), 2,2′-Azino-bis-(3-ethylbenzothiazoline-6-sulfonic acid) diammonium salt (ABTS), and 2,4,6-tripyridyl-C-triazine (TPTZ) were purchased from Shanghai Yuanye Bio-Technology Co. Ltd. (Shanghai, China). Anhydrous sodium carbonate, sodium nitrite, crystalline aluminum chloride, sodium hydroxide, potassium chloride, sodium acetate trihydrate, potassium persulfate, ferric chloride, ferrous sulfate, salicylic acid, and hydrogen peroxide were purchased from Tianjin Bodi Chemical Co. (Tianjin, China). HPLC-grade acetonitrile and methanol were obtained from Grace Company, Inc. (Houston, TX, USA). All the culture media used in the evaluation of the antimicrobial activity were acquired from Oxoid (Oxoid Limited, Thermo Fisher Scientific Inc., Waltham, MA, USA). The Millipore water purification system was used for the production ultrapure water.

### 3.3. Sample Preparation

The extraction method of peony seed kernel was according to Jin et al. with the following modifications [45]. The samples (2 g) were extracted with 15 mL of acidified methanol solution (1 M HCL in 80% methanol) for 24 h at 4 °C, and then the extract was centrifuged at 12,000 rpm for 10 min at 4 °C. The supernatants were collected, and then the remaining residue manufactured by the first extraction was dissolved in 12.5 mL of acidified methanol solution. The homogenate was placed in an ultrasound bath for 30 min at 25 °C and centrifuged at 12,000 rpm for 10 min at 4 °C. The residue was extracted twice in the same conditions. The supernatants of the two runs were combined for analysis.

### 3.4. Determination of Total Phenolic, Flavonoid, and Flavanol Content

The TPC in the seed kernel of peony was determined using the Folin–Ciocalteu reagent method, with the following modifications [46]. In short, 7.9 mL of distilled water, 0.1 mL of extract, and 0.5 mL of Folin–Ciocalteu reagent (10%) were added to centrifuge tube. After reaction for 5 min, 1.5 mL of Na_2_CO_3_ (20%, w/v) was added to the mixture. Then, the mixture was incubated for 2 h in the dark and the absorbance was measured at 765 nm using a UV-visible spectrophotometer (UV-1700, Shimadzu Crop., Kyoto, Japan). Values for TPC were expressed as milligrams of gallic acid equivalents (GAE) per g dry weight of the sample (mg GAE/g DW).

The TFC in the seed kernel of peony was measured by the method of Marghitas et al. [47]. Briefly, 1 mL of the extract, 0.3 mL of NaNO_2_ (0.5 M), and 0.3 mL of AlCl_3_ (0.3 M) were added in centrifuge tube. Next, 4 mL of NaOH (1 M) was added to the mixture after 5 min. The absorbance was measured at 510 nm. The TFOC was calculated based on the calibration curve of rutin. TFC was expressed as rutin equivalent per gram of dry weight (mg RE/g DW).

The TAC was measured as described by Jin et al. [46]. The mixture contained 5.0 mL of vanillin reagent (0.5 g of vanillin dissolved with 200 mL of 4% concentrated HCL in methanol) and 1 mL of the seed extract, and then, it was kept in the dark. The absorbance was read at a wavelength of 500 nm. TAC of the seed from peony was expressed as (+)-catechin equivalents per 100 g sample of dry weight (mg CE/100 g DW).

### 3.5. Qualitative and Quantitative Analysis of the Main Phytochemical Components

#### 3.5.1. HPLC Analysis

The proximate composition and phytochemical components in the peony seed kernel samples were analyzed on a Shimadzu HPLC system equipped with a diode array detector. The active compounds were separated on a Hibar RT LiChrospher column (SB-C18, 250 mm × 4.0 mm, 5 μm), using a gradient program with the two mobile phases of solvent A (water/acetic acid 99:1, v/v) and solvent B (acetonitrile), and the column temperature was maintained at 40 °C. The gradient program was as follows: 0–40 min, 5–40% B; 40–45 min, 40–100% B; and 45–60 min, 100% B. The flow rate was 1.0 mL/min. Each injection volume was 10 µL. The retention times and spectra were compared with those of pure standards. The wavelength range of the DAD detector was 190–400 nm. The peak assignments for all compounds were made by comparing their retention times and characteristic absorption spectra with those of the standards.

#### 3.5.2. Mass Spectrometry

Mass analysis was performed on an ACQUITY UPLC™ I-Class system coupled with a Xevo G2-C QTOF mass spectrometer (Waters CO., Palo Alto, CA, USA). The samples were analyzed in negative ion electrospray mode. MS parameters were as follows: capillary voltage, 2.5 kV; sampling cone voltage, 30 V; source temperature, 120 °C; desolvation temperature, 500 °C; cone gas flow, 100 L/h; and desolvation gas flow, 800 L/h. For mass accuracy, the LockSpray™ interface was used with a leucine enkephalin (555.2645 amu) solution for 0.2 s with an interval of 20 s to adjust the mass calibration of the instrument during analysis. Data were collected in the MS E mode. Each MS spectrum was obtained from 100 to 1500 Da. Data were processed and analyzed using UNIFI software. For positive matches, the mass error of the precursor was less than ±5 ppm.

### 3.6. Determining Tocopherols (α and γ Tocopherol) by HPLC

About 0.1 g dry power of seed kernel was extracted with 2 mL n-hexane. Then, the extracts were filtered into a sample vial through a 0.45 mm water membrane filter. Aliquots of this solution were subjected to HPLC analysis. The diode array detector scanned the samples from 200 to 400 nm, using the following standards: solvent used was acetic acid/water (98:2; v/v), the column temperature was maintained at 30 °C, and the flow rate was 1.2 mL/min. Each injection volume was 10 μL. The detection wavelengths for retinol and α and γ tocopherol were 325 and 298 nm, respectively.

### 3.7. Antioxidant Activity

Evaluation of the antioxidant capacity of the kernel of peony seed extracts was according to the DPPH method described by Brand et al. with slight modifications [48]. The chromatographic grade methanol was used for DPPH; concentration of extract was 25 mg/L. Then, 0.1 mL of extract (the dilution ratio is 1:19 used with methanol) from kernel and shell was added to 3.9 mL DPPH radical. At the same time, the same volume of methanol instead of the sample was used as a control to determine the maximum DPPH absorbance. Finally, the mixtures were kept in dark for 30 min for reaction, and the absorbance was measured at λ = 517 nm to determine the concentration of the remaining DPPH. The Trolox equivalents were used to express the results of this test, which represent per 100 g of dry weight.

The ABTS cation radical assay was carried out according to the method described by Re et al. with the following modifications [49]. The ABTS radical cation contains 5 mL ABTS solution (7 mM) and 88 μL potassium persulfate aqueous solution (2.45 mM). Then, the mixture was stood for 12 h in the dark before use. When use the mixture diluted with methanol, make sure that the absorption at λ = 732 nm is 0.70 ± 0.02. After the formation of ABTS solution, 0.1 mL of extract (diluted with methanol at 1:20) from kernel and shell was mixed with 3.9 mL ABTS radical cation solution, and then the absorbance was measured at λ = 732 nm after reaction in a dark room (for exactly 8 min). The Trolox equivalents were used to express the results of this test, which represent per 100 g of dry weight.

The scavenging activity of ferric reducing antioxidant power (FRAP) radical was determined as described by Benzie et al. [50] with slight modifications. Precisely, 0.3 M sodium acetate buffer (PH 3.6), 20 mM FeCl_3_·6H_2_O, and 10 mM TPTZ solution were mixed with 40 mM HCL in a volume ratio of 10:1:1 to prepare the FRAP solution. Then, 0.2 mL of peony seed extract and 1.8 mL of working FRAP solution were mixed. The mixture was warmed to 37 °C for 10 min, and reading was taken at λ = 593 nm after 10 min of incubation. The Trolox equivalents were used to express the results of this test, which represent per 100 g of dry weight.

The hydroxyl radical scavenging activity (HRSA) was determined using the method proposed by Halliwell et al. [51]. The hydroxyl radicals were generated through the following system: 0.8 mL of distilled water, 0.2 mL of FeSO_4_ (9 mM), 0.2 mL of salicylic acid (9 mM), and 100 μL of the sample were added in sequence. Then, during incubation for 30 min at 37 °C, 9 μL H_2_O_2_ (0.15%) was added to the mixture. The absorbance was measured at 536 nm. The results were expressed as inhibition relative to a control test (without the sample).

### 3.8. Determination of Antibacterial and Antifungal Activities

The seed kernel extracts were used to test against four pathogenic bacterial strains and two pathogenic fungi: *Listeria monocytogenes* (Gram-positive ATCC 7644), *Bacillus subtilis* (Gram-positive ATCC 6633), *Proteus vulgaris* (Gram-negative ATCC 8427), *Salmonella typhimurium* (ATCC 13311), *Candida albicans* (Fungi ATCC 6645), and *Microsporum gypseum* (Fungi ATCC 14683).

The method about minimum inhibitory concentration (MIC) of the extracts was carried out by method of Zhang et al. with the following modifications, i.e., in a microtiter plate (96 wells) [7]. The extracts were analyzed using a two-fold dilution series prepared in dimethyl sulfoxide (DMSO) (4%). The extracts were divided into six gradients, ranging from 1 to 0.03 mg/mL. Nutrient broth and Sabouraud dextrose agar were used for bacteria and fungi, respectively. First, the suspension at 10^6^ CFU mL^−1^ (0.5 McFarland Scale) was prepared for microorganisms. Each microwell contained 100 μL of extracts and 100 μL of cell suspension. Then, the plates loaded with bacteria and fungi were incubated at 37 °C and 28 °C for 24 and 48 h, respectively. Microbial growth was evaluated by recording values at a wavelength of 620 nm. The DMSO was used as a blank control only for the well-containing bacteria or fungi in the adequate medium.

Inhibition zone results (IZR) for different extracts were evaluated by following the method explained by Rojas et al. [52]. Immediately after autoclaving, the substrate was cooled at about 45–50 °C by keeping it at room temperature. Then, the substrate was poured into flat-bottomed Petri dishes (90 mm in diameter and depth of almost 4 mm). After solidification, 0.1 mL of the bacterial or fungi was evenly spread on the surface of the solidified agar by using sterile cotton. The four uniform paper discs (6 mm in diameter) were placed on the surface of the solidified agar. Each paper disc was soaked with different extracts (1 mg/mL). Then, the plates were incubated for 24 h at 37 °C for bacteria or 48 h at 28 °C for fungi. DMSO was used as negative control.

### 3.9. Data Analysis

All experiments were carried out in triplicate. The results were analyzed and expressed as the mean ± standard deviation (SD). A two-tailed Pearson’s correlation test was performed to determine the correlations between phytochemical components and bioactivity activities. The significance of the differences between samples at *p* ≤ 0.05 was determined by analysis of variance (ANOVA) and Duncan’s multiple range tests. In terms of the equipartition principle, the hierarchical cluster analysis (HCA) was performed to group the populations with Ward’s method. Temperature data comes from China Meteorological Administration. All analyses were performed using the SPSS software (Version 21.0 for Windows, IBM, New York, NY, USA).

## 4. Conclusions

The results showed that the TPC, TFC, TAC, and the antioxidant activity varied among different peony species, and they were much higher in the seeds of *P. delavayi*, *P. obovata.* subsp*. obovata,* and *P. rockii.* subsp*. rockii* than in those of the other samples; therefore, these species could be valuable sources of antioxidants for value-added food product development. In terms of individual chemical compounds, large differences between populations were observed, and most samples were rich in procyanidin B2, benzoic acid, paeoniflorin, and oxypaeoniflora. *P. mairei* was grouped individually into a group via hierarchical cluster analysis according to its relatively highest monoterpene glycosides and TPC content. Overall, the seeds from *P. mairei, P. delavayi*, *P. obovata.* subsp*. obovata,* and *P. rockii.* subsp*. rockii* could be valuable sources to the food and pharmaceutical industries. This work has provided a basis for the development and utilization of seeds for the selection of wild peony species of dietary interest.

## Figures and Tables

**Table 1:** The 25 common metabolites identified in the seeds of peony species using HPLC–UV–MS.

Peak No.	Assigned Identity	*t_R_* (min)	Molecular Formula	UV λ Max (nm)	[M − H]^−^ *m*/*z*		[M + HCOO]^−^ *m*/*z*	Other Fragment Ions of [M − H]^−^ at Low Energy
					Mean Measured Mass (Da)	Theoretical Exact Mass (Da)		
1	Procyanidin B1	3.15	C_30_H_26_O_12_	275	577.1352	577.1962		451,425,407,288
2	Oxypaeoniflora	3.26	C_23_H_28_O_12_	200,258	495.1501	495.1508	531.1364	151,197
3	Procyanidin B2	3.4	C_30_H_26_O_12_	275	577.1968	577.1263		407,425,407,289
4	Rutin	3.65	C_27_H_30_O_16_	355,255	609.1431	609.1435		300
5	Albiflorin	3.78	C_23_H_28_O_11_	195,230	479.1552	479.1553	525.1663	324,255,225,175
6	Paeoniflorin	3.85	C_23_H_28_O_11_	196,265,290	459. 1498	459.1503	525.1536	449,327,165,121
7	Eriodictyol	3.93	C_15_H_12_O_6_	255	288.2540	288.2541		287,151,135
8	Quercetin 3-β-D-glucoside	3.95	C_21_H_18_O_13_	255,350	625.1404	625.1405		463,300
9	Cyanidin 3-arabinoside	3.99	C_21_H_21_ClO_11_	280,515	454.8063	454.8067	419.1347	326
10	Methyl Gallate	4.01	C_8_H_8_O_5_	220,271	183.0299	183.0294		124
11	Cyanidin-3-galactoside chloride	4.05	C_21_H_21_ClO_11_	280,515	484.8376	484.8378	465.1214	343
12	Syringate	4.24	C_9_H_10_O_5_	282	198.1638	198.1702		181,153,121
13	Apigenin-7-O-glucuronide	4.27	C_21_H_18_O_11_	231,265,335	446.3609	446.3613		269,175
14	Phlorizin	4.42	C_12_H_24_O_10_	435,273	436.4120	436.4123		315,273
15	(+)-Dihydromyricetin	4.82	C15H12O8	292	321.0601	321.0541		303,153,149
16	Resveratrol	5.18	C_14_H_12_O_3_	216,318	228.2031	228.2032	229.2162	227,143,185,159
17	Benzoyl paeoniflorin	5.33	C_30_H_32_O_13_	195,230,272	599.1766	599.1765	629.1856	447,431,281,137
18	Luteolin	5.35	C_15_H_10_O_6_	236,266	286.2311	286.2314		175,133
19	Quercetin	5.41	C_21_H_20_O_11_	249,372	302.0352	302.0348		275
20	Benzoic acid	5.46	C_6_H_5_COOH	201,234	121.0321	121.029		77
21	Salicylic acid	5.65	C_7_H_6_O_3_	300	138.1198	138.121		92,65
22	Apigenin	5.91	C_15_H_10_O_5_	340	270.1436	270.2214		117,107,121
23	Kaempferol	5.98	C_15_H_10_O_6_	200,260,360	287.0549	287.0422		259,241,213
24	p-coumalic acid	6.16	C_6_H_4_O_4_	335	164.1614	164.1617		119,95
25	Paeonol	7.12	C_9_H_10_O_3_	220,235,272,316	165.0549	165.0552		149,121

**Table 2:** Geographic conditions of original habitats of six groups.

Group	Longitude E (°)	Latitude N (°)	Elevation (m)
1	85.33–106.36	28.31–47.13	1897–3598
2	103.45	31.58	2176
3	99.58–118.52	26–41.63	1216–3345
4	86.58–128.01 121.11w	29.73–48.42	462–3103
5	106.11	33.25	1765
6	109.17	33.0	1623

**Table 3:** Geographic conditions of original habitats of 21 populations.

Population	Taxon	Sample Locality	Longitude E (°) and Latitude N (°)	Elevation (m)	Sect.
P1	*Paeonia. lactiflora*	Feng County, Shaanxi	106.57/34.14	1661	Sect. *Paeonia*
P2	*P. lactiflora*	Lveyang county, Shaanxi	106.21/33.22	1685	Sect. *Paeonia*
P3	*P. lactiflora*	Ning county, Nei Mongol	118.52/41.63	1216	Sect. *Paeonia*
P4	*P. anomala* subsp*. veitchii*	Wudu district, Gansu	102.19/31.51	2771	Sect. *Paeonia*
P5	*P. anomala* subsp*. veitchii*	Feng County, Shaanxi	106.36/34.10	1897	Sect. *Paeonia*
P6	*P. anomala* subsp*. veitchii*	Lintao county, Gansu	104.0/35.42	2809	Sect. *Paeonia*
P7	*P. anomala* subsp*. anomala*	Jimunai county, Xinjiang	85.55/47.13	1955	Sect. *Paeonia*
P8	*P. anomala* subsp. *anomala*	Kanas, Xinjiang	86.58/48.42	1658	Sect. *Paeonia*
P9	*P. emodi*	Jilong country, Tibet	85.33/28.31	2348	Sect. *Paeonia*
P10	*P. stemiana*	Bomi County, Tibet	96.05/29.73	3103	Sect. *Paeonia*
P11	*P. obovata* subsp*. willmottiae*	Lueyang county, Shaanxi	106.11/33.25	1765	Sect. *Paeonia*
P12	*P. obovata.* subsp*. obovata*	Shangzhi city, Heilongjiang	128.01/45.21	251	Sect. *Paeonia*
P13	*P. obovata.* subsp*. obovata*	Jiaohe city, Jilin	127.44/43.57	462	Sect. *Paeonia*
P14	*P. mairei*	Xunyang County, Shaanxi	109.17/33.0	1623	Sect. *Paeonia*
P15	*P. brownii*	San Benito, California	121.11w/36.32	533	Sect. *Onaepia*
P16	*P. delavayi*	Shangri-La, Yunnan	99.58/27.97	3345	Sect. *Moutan*
P17	*P. delavayi*	Lijiang city, Yunnan	100.17/26.80	3015	Sect. *Moutan*
P18	*P. decomposita*	Li country, Sichuan	103.45/31.58	2176	Sect. *Moutan*
P19	*P. rockii.* subsp. *rockii*	Shennongjia Forestry district, Hubei	110.69/31.77	1933	Sect. *Moutan*
P20	*P. qiui*	Xunyang county, Shaanxi	109.24/32.59	1272	Sect. *Moutan*
P21	*P. ludlowii*	Linzhi city, Tibet	94.17/29.15	3598	Sect. *Moutan*

## Supplementary Materials

The following are available online at https://www.mdpi.com/2223-7747/9/6/729/s1, Table S1. Antimicrobial activities (MIC mg/ml) of seeds extracts from different species. Table S2. Antimicrobial activities (IZR mm) of seeds extracts from different species.
