# Supplementary material for: Insights into the Phytochemical Composition and Bioactivities of Seeds from Wild Peony Species
Source: Plants (Basel). 2020 Jun 9;9(6):729. doi: 10.3390/plants9060729 (PMC7356631; doi:10.3390/plants9060729)
Supplement: Supplementary file 1 [file plants-09-00729-s001.pdf]

plants

MDPI

Supplementary Materials:

Table S1. Antimicrobial activities (MIC mg/ml) of seeds extracts from different species.

	Listeria monocytogenes	*Bacillus subtilis*	*Proteus vulgaris*	*Salmonella Typhimurium*	*Candida albicans*	*Microsporum gypseum*
P1	0.06 ± 0.02	0.12 ± 0.02	0.25 ± 0.02	0.50 ± 0.04	0.25 ± 0.02	0.25 ± 0.02
P2	0.25 ± 0.02	0.12 ± 0.02	0.12 ± 0.02	0.50 ± 0.02	0.25 ± 0.02	0.25 ± 0.04
P3	0.25 ± 0.02	0.25 ± 0.04	0.5 ± 0.06	0.50 ± 0.06	0.25 ± 0.02	0.25 ± 0.02
P4	0.50 ± 0.06	0.12 ± 0.02	0.25 ± 0.02	0.25 ± 0.02	0.25 ± 0.02	2.00 ± 0.08
P5	0.12 ± 0.02	0.50 ± 0.06	0.50 ± 0.04	0.50 ± 0.04	2.00 ± 0.02	0.25 ± 0.02
P6	0.25 ± 0.02	0.25 ± 0.02	0.12 ± 0.02	0.50 ± 0.02	2.00 ± 0.02	0.25 ± 0.04
P7	0.25 ± 0.04	0.12 ± 0.02	0.50 ± 0.02	0.50 ± 0.02	0.50 ± 0.02	0.25 ± 0.02
P8	0.25 ± 0.02	0.25 ± 0.02	0.25 ± 0.04	0.50 ± 0.06	2.00 ± 0.12	0.50 ± 0.06
P9	0.12 ± 0.04	0.12 ± 0.02	0.50 ± 0.02	0.25 ± 0.02	0.50 ± 0.02	2.00 ± 0.14
P10	0.03 ± 0.00	0.12 ± 0.04	0.25 ± 0.04	0.50 ± 0.04	2.00 ± 0.08	0.5 ± 0.02
P11	0.50 ± 0.04	0.25 ± 0.06	0.50 ± 0.02	0.25 ± 0.02	0.50 ± 0.06	0.50 ± 0.02
P12	0.12 ± 0.02	0.12 ± 0.02	0.25 ± 0.02	0.25 ± 0.02	0.50 ± 0.04	0.25 ± 0.02
P13	0.06 ± 0.02	0.50 ± 0.06	2.00 ± 0.24	0.50 ± 0.02	2.00 ± 0.24	0.25 ± 0.02
P14	0.25 ± 0.06	0.06 ± 0.02	0.50 ± 0.04	0.50 ± 0.02	0.25 ± 0.04	0.50 ± 0.02
P15	0.50 ± 0.04	0.25 ± 0.02	0.50 ± 0.06	0.25 ± 0.02	0.50 ± 0.02	0.25 ± 0.02
P16	0.25 ± 0.02	0.12 ± 0.02	0.25 ± 0.02	0.25 ± 0.02	0.25 ± 0.02	0.50 ± 0.02
P17	0.12 ± 0.02	0.25 ± 0.02	1.00 ± 0.06	1.00 ± 0.12	0.25 ± 0.04	0.25 ± 0.02
P18	0.25 ± 0.04	0.12 ± 0.02	0.25 ± 0.02	0.50 ± 0.02	0.25 ± 0.06	0.25 ± 0.02
P19	0.25 ± 0.02	0.25 ± 0.02	0.50 ± 0.02	0.50 ± 0.04	1.00 ± 0.12	1.00 ± 0.08
P20	0.50 ± 0.02	0.12 ± 0.02	0.50 ± 0.04	0.50 ± 0.02	0.25 ± 0.04	0.25 ± 0.02
P21	0.50 ± 0.02	0.06 ± 0.00	0.12 ± 0.02	0.50 ± 0.06	0.25 ± 0.04	0.25 ± 0.02

Table S2. Antimicrobial activities (IZR mm) of seeds extracts from different species.

	Listeria monocytogenes	*Bacillus subtilis*	*Proteus vulgaris*	*Salmonella Typhimurium*	*Candida albicans*	*Microsporum gypseum*
P1	16.54 ± 0.99	10.89 ± 0.61	15.85 ± 0.99	10.26 ± 0.57	10.62 ± 1.01	11.12 ± 0.77
P2	12.14 ± 1.33	10.62 ± 1.16	13.12 ± 1.88	10.58 ± 1.16	11.89 ± 0.19	10.69 ± 0.27
P3	11.09 ± 0.99	13.36 ± 1.01	16.54 ± 1.25	11.69 ± 0.88	9.35 ± 3.55	11.23 ± 0.43
P4	10.95 ± 0.65	16.21 ± 0.97	14.77 ± 1.06	14.62 ± 0.87	9.65 ± 0.82	10.98 ± 0.98
P5	10.32 ± 0.61	8.95 ± 0.53	17.14 ± 1.02	10.05 ± 0.6	7.12 ± 0.71	10.23 ± 1.74
P6	14.15 ± 0.84	8.57 ± 0.5	16.45 ± 0.97	9.68 ± 0.57	6.98 ± 0.44	10.65 ± 0.69
P7	9.89 ± 0.89	15.79 ± 1.42	15.14 ± 1.36	11.69 ± 1.05	10.06 ± 0.58	10.81 ± 0.62
P8	14.56 ± 0.87	13.56 ± 0.15	15.31 ± 0.17	10.75 ± 0.12	7.03 ± 0.51	9.86 ± 0.78
P9	11.12 ± 0.55	10.26 ± 0.51	15.29 ± 0.76	12.12 ± 0.6	9.89 ± 0.36	7.24 ± 0.28
P10	13.65 ± 0.81	16.52 ± 0.26	16.71 ± 0.26	10.03 ± 0.16	7.65 ± 0.61	10.03 ± 0.98
P11	10.23 ± 1.02	12.09 ± 1.08	16.52 ± 1.48	11.26 ± 1.01	9.16 ± 0.73	9.78 ± 0.83
P12	16.45 ± 0.32	16.14 ± 0.32	16.21 ± 0.32	11.02 ± 0.22	9.89 ± 0.79	10.56 ± 0.8
P13	9.21 ± 0.59	10.91 ± 0.72	16.01 ± 1.05	11.65 ± 0.76	7.02 ± 0.53	10.78 ± 0.78
P14	16.21 ± 2.11	17.21 ± 2.23	14.23 ± 1.84	10.13 ± 1.31	11.03 ± 0.88	9.96 ± 0.23
P15	11.23 ± 0.25	13.16 ± 0.3	16.79 ± 0.38	14.36 ± 0.33	9.62 ± 0.77	11.06 ± 0.8
P16	14.58 ± 0.87	16.89 ± 1.01	17.14 ± 1.02	15.02 ± 0.9	10.67 ± 1.17	9.89 ± 1.48
P17	15.79 ± 0.64	13.95 ± 0.57	15.69 ± 0.64	15.98 ± 0.65	10.92 ± 0.79	11.12 ± 1.06
P18	12.78 ± 1.91	15.04 ± 2.25	11.04 ± 1.65	9.32 ± 1.39	9.93 ± 0.65	10.23 ± 0.66
P19	12.12 ± 0.24	13.26 ± 0.26	12.68 ± 0.25	9.98 ± 0.19	10.35 ± 0.75	9.06 ± 0.65
P20	10.16 ± 0.61	15.69 ± 0.94	16.01 ± 0.96	10.96 ± 0.65	10.98 ± 0.37	10.26 ± 0.55
P21	12.01 ± 0.72	16.81 ± 1.22	15.19 ± 1.1	10.03 ± 0.73	10.71 ± 0.19	11.03 ± 0.2
